# Effects of a Walking Exercise-Focused Health Promotion Program for Middle-Aged Women in the Korean Community

**DOI:** 10.3390/ijerph192214947

**Published:** 2022-11-13

**Authors:** Soojeong Yang, Hyunlye Kim

**Affiliations:** 1 Department of Nursing, Chosun Nursing College, Gwangju 61452, Korea; 2 Department of Nursing, College of Medicine, Chosun University, Gwangju 61452, Korea

**Keywords:** middle aged, health promotion, walking, waist circumference, psychological stress

## Abstract

We assessed the effectiveness of a walking exercise-focused health promotion program based on an information–motivation–behavioral skills model. This intervention study employed a non-equivalent control group pre-test/post-test design. We recruited 44 middle-aged women (22 per group) who visited two health check-up centers in G city of South Korea. The intervention included information (health education), personal motivation (pedometer monitoring, setting goals, and keeping an exercise log), social motivation (group discussion and support, telephone counseling), and behavioral skills (walking exercise) components. Ten sessions of this program involved three face-to-face and seven online interactions via mobile instant messaging. As primary outcomes (health behavior), physical activity level and health-promoting lifestyle were measured by standardized scales. As secondary outcomes (health status), physiological indicators by body measurements and perceived stress by a scale were produced. We used the chi-squared test, independent *t*-test, and Mann–Whitney U test for the analysis. After the intervention, in the experimental group, the level of physical activity (Z = −2.065, *p* = 0.039) and health-promoting lifestyle improved (t = 3.344, *p* = 0.002), and both waist circumference (t = −4.328, *p* < 0.001) and perceived stress (t =−3.578, *p* < 0.001) decreased. In conclusion, our theory-based intervention has advantages in terms of high standardization potential, high availability, and improvement of health behavior and health status. In future, this approach will be useful for devising interventions that meet the health needs of people who are concerned about quality of life in the second half of life.

## 1. Introduction

In the midst of the health care challenges of an extending lifespan and an aging population, there is growing interest in staying healthy throughout life [1]. According to data from Statistics Korea in 2020, life expectancy was 83.5 years, which is about 21 years higher than in 1970 (62.3 years) [2]. In particular, the life expectancy of women is now 86.5 years, which is 6 years longer than that of men (80.5 years) [2]. These data suggest the importance of preparing for later life.

Midlife, the period in which one transitions from the first to the second half of the life course, is pivotal for health management [3]. Middle-aged adults may place their physical and mental health at risk by making various changes based on re-evaluation of the past; this is often characterized as a “midlife crisis” [3,4], which is associated with unhealthy behavior and reduced life satisfaction [5]. Middle age, along with age-related physical decline, leads to changes in heart health and cardiovascular risk profile [6]. It is also associated with the onset of functional limitations, and the transition to disability may occur with the risk of osteoporosis and concomitant fractures [6]. In particular, middle-aged women experience various physical and psychological symptoms as they go through menopause, which negatively affect quality of life [7,8]. However, middle age also offers new opportunities and challenges [9]. It is possible to balance gains and losses, and meaningfully connect one’s previous and current life circumstances [9]. Middle-aged women may require assistance to improve their health so that the second half of the life course is of a high quality.

Health management during middle age determines the quality of the rest of one’s life, and effective health-promoting measures are important during this period [10]. In a cohort study analyzing the 10-year outcomes of health promotion programs (n = 10,248; mean age 41.2 ± 10.8 years; female 68.1%), the behaviors most important for good health were a low-fat diet, aerobic exercise, smoking cessation, and sufficient sleep [11]. Previous studies have reported that interventions targeting health promotion have positive effects on the health behaviors, health risk factors, and physical/mental health status of middle-aged women. In an intervention study for postmenopausal women, health education with a focus on health-promoting lifestyle modifications was effective in enhancing their adherence to health-promoting behaviors and improving menopause symptoms, anthropometric adiposity indices, cardiovascular disease risk indicators, hand grip strength, and gait speed [12]. Health education, focused on a health-promoting lifestyle, has also been effective in improving the overall quality of life of postmenopausal women [13]. A study on middle-aged Korean women showed that a health-promoting lifestyle had a direct mediating effect on the relationship between menopausal symptoms and depression [14]. These findings show the benefits of a health promotion approach to improving the health behavior and health status of middle-aged women.

Walking exercise has been suggested as an effective and safe health promotion activity with high adherence and wide-ranging health benefits [15]. In a randomized controlled trial of an urban forest-walking program for office workers, physical activity level, health-promoting behavior, and quality of life all improved [16]. In a study including a large group of middle-aged South Korean women, regular walking was associated with reductions in perceived stress and depression, and a greater capacity for activity [17]. Regular walking exerted positive effects on body weight, fat mass, blood biochemical markers, and physical activity parameters in middle-aged women [18,19]. Therefore, health promotion interventions that include walking may improve the health behavior and health status of middle-aged women.

Physical inactivity is one of the major modifiable risk factors for mortality and is a key target of interventions aimed at promoting health [20]. Compared to their physically less active counterparts, physically active individuals across all ethnicities and ages exhibit higher levels of cardiorespiratory fitness, health, and well-being, and have lower risks of various chronic medical conditions [20]. A cross-sectional epidemiologic study of a UK population found that physically active people had better mental health in terms of symptoms of depression/anxiety and mental well-being [21]. In a meta-analysis, postmenopausal women had higher cardiovascular disease risk factors, morbidity, and mortality than women of reproductive age, and physical activity lowered the risk [22]. Therefore, we selected the level of physical activity as a target outcome based on health promotion interventions.

We adopted the information–motivation–behavioral skills (IMB) model as a theoretical basis for designing effective health promotion interventions [23]. This psychological model considers information, motivation, and behavioral skills as fundamental determinants of health behavior [23]. The IMB model has been useful in devising health education or training programs to improve health behaviors [24,25,26,27,28]. In addition, a meta-analysis study of electronic health interventions to promote physical activity in older people suggested this as an effective and safe intervention strategy [29]. In particular, instant messaging and social networks, which allow users to exchange instant messages online or via mobile devices, are gaining traction in the field of health research [30]. Additionally, to apply electronic health interventions effectively to older adults, consideration should be given to reducing the barriers they face, and utilizing facilitators [31]. Therefore, we additionally adopted the online method using mobile instant messengers (MIM), and used the offline method in parallel to reflect the barriers and facilitation factors.

This study aimed to provide a health promotion program centered on walking exercise based on an information–motivation–behavioral skills model for middle-aged women, and to confirm its effect on health behavior and health status. The hypotheses of this study were as follows:

The experimental group will have an increased level of physical activity.

The experimental group will have an improved health-promoting lifestyle.

The experimental group will have improved physiological indicators, such as body mass index, waist circumference, and blood pressure.

The experimental group will have lower perceived stress.

## 2. Conceptual Framework

**Figure 1:**
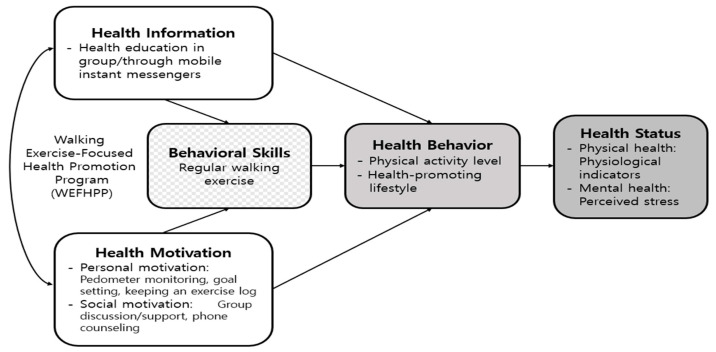
Conceptual framework of this study.

Our health promotion intervention was based on the IMB model of Fisher et al. (2006) [23]. We designed intervention strategies that correspond to the main components of the IMB model, to reinforce healthy behaviors as follows: information (health education), personal motivation (pedometer monitoring, setting goals, and keeping an exercise log), social motivation (group discussion and support, telephone counseling), and behavioral skills (walking exercise) (Figure 1). We created educational materials delivered weekly through group training or instant messaging throughout the program. Health motivation was divided into personal and social domains according to the IMB model [23]. To reinforce personal motivation, we gave each participant a pedometer and asked them to keep an exercise log. In terms of social motivation, we provided emotional support and helped set goals via telephone counselling and group training. Behavioral skills for adhering to healthy behaviors are assumed to determine whether even well-informed and motivated individuals can maintain healthy behaviors [23]. In this study, regular walking exercise was selected as a behavioral skill for health behavior. Primary and secondary outcome variables were established to identify the effects of interventions, including health information, health motivation, and behavioral skill components. Primarily, this walking exercise-centered health promotion program was expected to improve the health behavior, identified by physical activity level and health-promoting lifestyle. Secondarily, it was hypothesized that the experimental group after the intervention would show improvements in physiological indicators and perceived stress.

## 3. Materials and Methods

### 3.1. Study Design

This intervention study used a non-equivalent control group pre-/post-test design to explore the effects of a health promotion program for middle-aged women.

### 3.2. Ethical Considerations

The study began only after approval had been obtained from the Institutional Review Board (approval no. 2-1041055-AB-N-01-2017-0034). The purpose, procedure, duration, research methods, anticipated results, and applications of the study were fully explained to all participants in advance, and they were informed that they were free to leave the study at any time. The written informed consent forms and completed questionnaires were kept in a safe that was accessible only to the researchers. At the end of the program, we rewarded the experimental group for participation (pedometer and small gift certificate), and provided the control group with a pedometer and health education.

### 3.3. Study Sample

The target population were Korean women aged 40–59 years in the community (8,285,456 people as of 2021) [32]. The inclusion criteria were as follows: middle-aged women who agreed to participate in the study; those able to participate in the program for 10 weeks, and who had no physical impairments, were able to read/write/communicate, had no previous experience in walking exercise programs, and had not participated in any other physical activity programs at the time of the program. The exclusion criteria were as follows: unstable blood pressure control; did not engage in walking for more than 2 weeks in the experimental group or participated in other physical activity programs in the control group.

Participants were recruited via posters when they visited one of two health check-up centers in G city. The optimal sample size for *t*-test analysis was calculated using G*Power software (ver. 3.1.2) [33]. The effect size was based on a prior study of the effects of health-promoting programs (education and exercise) on middle-aged women [34]. For a one-sided significance level of 0.05, power (1−β) of 0.80, and effect size of 0.80, the experimental and control groups each required 21 subjects. Assuming a dropout rate of about 18% (as in a previous walking program) [35], we recruited 25 experimental and 26 control subjects. We assigned an experimental group and a control group via a randomization method. After notifying the participants in advance that they would be randomized to either the experimental group or the control group, a coin toss was performed to determine the group assignment for each participant. The coin tossing was stopped when the target number of people in either group was met. The remaining applicants were placed in an unpopulated group. Four participants dropped out because of personal reasons (hospitalization or employment-related) and three were found to have provided inappropriate questionnaire responses so were excluded after study completion. Finally, data from 22 participants in each group were analyzed.

### 3.4. Data Collection

Data were collected from September to November 2017. The visit times of the participants were adjusted to prevent contact between the experimental and control groups. The intervention period was early autumn (at which time outdoor activities are not affected by excessively cold or hot conditions). A pre-test and post-test were conducted to confirm the change in the effect index before and after the intervention. The data collection methods used were structured questionnaires and body measurements for the experimental and control groups. For personal motivation, we provided participants with a pedometer, which they wore around their waist. Each pedometer was equipped with a function to check the number of steps and to estimate calories burned by entering the user’s height, weight, and average stride length.

### 3.5. Research Progress

#### 3.5.1. Pretesting

Pretesting involved a self-reported structured questionnaire on general characteristics, physical activity, health-promoting behaviors, and perceived stress. Participants were asked to overnight fast after the previous evening’s dinner and visit the center in the morning for body composition measurements. Height, weight, waist circumference, and blood pressure were measured by a trained research assistant who was a nurse with more than 3 years of experience at the check-up centers. In this case, an automatic height/weight scale (BSM330, Biospace, Seoul, Korea), a soft string ruler, and an automatic blood pressure monitor for adults (JPN600, Omron, Japan) were used. The experimental group was asked to wear a pedometer from the time they woke up in the morning to just before going to bed, record the number of steps per week and calories burned per week, and report it via instant messaging.

#### 3.5.2. Treatment: Walking Exercise-Focused Health Promotion Program (WEFHPP)

The WEFHPP was devised by modifying the walking exercise program developed for office workers by Choi (2012) for middle-aged women [36], based on the IMB model. The structure and content of each session of this program are shown in Table 1. A total of 10 sessions of the WEFHPP consisted of 3 face-to-face sessions (Sessions 1, 6, and 10) and 7 online sessions (Sessions 2–5 and 7–9). The offline session was held in the health examination center room by the first author, and the online session was conducted through instant messages. One session was held each week for 10 weeks. Nine health education sessions (conducted face-to-face or via instant messaging) were also completed from Sessions 1 to 9. We sent instant messages weekly during the online session. The messages were about how to walk properly, personalized exercise method and intensity, stretching, nutrition, how to manage stress, and how to increase daily activity level. Additional materials in the form of documents and videos were attached depending on the subject. To maintain personal motivation, participants were asked to monitor the numbers of steps taken and calories burned using the pedometer, and to create daily exercise logs. The exercise log included records of exercise content (walking, jogging, aerobics, etc.), exercise steps, exercise time, and total number of steps. Participants were also asked to set a weekly walking goal and self-evaluate it. For social motivation, three group discussions, two individual telephone counseling sessions, and continuous group support were provided. The content validity of the program was 0.95, as determined by six experts comprising two internal medicine specialists, one family medicine specialist, and three nursing professors.

#### 3.5.3. Post-Testing

Post-testing was performed during week 10 (final intervention session). The pretest questionnaires and physical measurements were repeated.

### 3.6. Instruments

#### 3.6.1. Physical Activity Level

We used the Korean version of the International Physical Activity Questionnaire Short Form (IPAQ-SF) [37]. In a study on Korean adults, the Spearman Rho coefficients and kappa values for test–retest reliability for seven components (days and minutes of vigorous, moderate, and walking exercise, and minutes spent sitting) were 0.43~0.65 and 0.37~0.62, respectively [38]. The Spearman Rho coefficient for all items in this study was 0.77. The total physical activity level was obtained by summing the walking, moderate, and vigorous activity times over the past 7 days. Questionnaire data were converted into metabolic equivalent task minutes per week (MET∙min/wk) using the average MET score for each type of activity. The weighted physical activity score was calculated as follows: duration × frequency per week × MET intensity (walking = 3.3 METs, moderate physical activity = 4.0 METs, vigorous physical activity = 8.0 METs).

#### 3.6.2. Health-Promoting Lifestyle

We used the Korean translation of the Health-Promoting Lifestyle Profile–II (HPLP-II) questionnaire introduced by Walker et al. [39,40,41]. The HPLP-II has 52 items and six categories: nutrition, physical activity, health responsibility, stress management, interpersonal relations, and spiritual growth. Items are scored on a four-point scale (1 = never, 2 = sometimes, 3 = often, 4 = routinely), and total scores range from 52 to 208. A higher score indicates a healthier lifestyle. The reliability (Cronbach’s α) value was 0.93 in the study of Hwang [39] and 0.92 in the present study.

#### 3.6.3. Physiological Indicators

Body mass index (BMI, weight in kilograms divided by height in meters squared), waist circumference, and blood pressure were measured, as in previous walking programs [16,35]. Relevant pre- and post-testing values were measured and compared by trained nurses who were blinded to group assignments. All body measurements were performed using the same instruments, at the same time of day and location. Blood pressure was measured twice on the upper arm at the height of the heart, 10 min apart, after each participant had rested in a chair for at least 10 min. The average value was used in the analysis.

#### 3.6.4. Perceived Stress

We used the validated 10-item Korean version of the Perceived Stress Scale (PSS) [42,43]. Items were scored on a five-point scale (0 = never, 1 = almost never, 2 = sometimes, 3 = fairly often, 4 = very often), and total scores ranged from 0 to 40. A higher score indicates a higher level of perceived stress. In Korea, the Cronbach’s α values (internal consistency) were 0.77 and 0.74 for negative and positive items, respectively [43]. In this study, the Cronbach’s α for all questions was 0.73.

### 3.7. Data Analyses

The data were statistically analyzed using SPSS for Windows software (ver. 22.0) and are presented as frequencies, percentages, and means with standard deviations. The Kolmogorov–Smirnov test was initially used to determine whether variables were normally distributed; a non-parametric test was performed if this was not the case. The independent *t*-test and the Mann–Whitney U test were employed to compare homogeneities between the experimental and control groups. The internal consistency (reliability) of the measurement tools was evaluated by calculating Cronbach’s α values and Spearman–Brown coefficients.

## 4. Results

### 4.1. Homogeneity Test

According to the pre-test data, the two groups did not differ significantly in terms of general characteristics or physical measurements, i.e., they were homogeneous (Table 2). Physical activity level, health-promoting behaviors (exercise and nutrition), and diastolic blood pressure were not normally distributed, so those data were analyzed using the Mann–Whitney U test.

### 4.2. Hypothesis Testing: Differences between Groups

Compared with the control group, the experimental group exhibited a significantly increased physical activity level (*p* = 0.035), improved health-promoting lifestyle (*p* = 0.002), decreased waist circumference (*p* < 0.001), and reduced perceived stress (*p* < 0.001) (Table 3). Among the subcategories of health-promoting lifestyles, there were between-group differences in physical activity (*p* < 0.001), nutrition (*p* = 0.006), and stress management (*p* = 0.012).

## 5. Discussion

Our health promotion intervention (WEFHPP) was designed to enhance the healthy lives of middle-aged women. This program had a significant effect on improving physical activity levels, a health-promoting lifestyle, waist circumference, and perceived stress. Previous health intervention studies using the IMB model [24,25,26,27,28] also reported positive effects in improving health behavior and health status. Therefore, the IMB model is highly recommended as a theoretical basis for health promotion programs.

The WEFHPP included 7 online sessions using MIM out of a total of 10 sessions, and was mainly used as a means of health education and group support. Instant messaging has been applied as a safe and effective means in the field of health care education and intervention [44,45,46,47]. A systematic review of mobile apps for cancer pain management found that instant messaging interventions were highly effective for controlling pain [48]. Our study demonstrates that e-health strategies via instant messaging are beneficial in terms of their safe adoption, even in a pandemic situation, and their effectiveness as a delivery mode of exercise programs or health promotion interventions for middle-aged women.

The WEFHPP was found to improve overall physical activity level (*p* = 0.035) and health-promoting lifestyle (*p* = 0.002) as a primary outcome variable. In particular, among the sub-concepts of health-promoting lifestyle, there were statistically significant improvements in physical activity, nutrition, and stress management. By contrast, no significant group difference was found in the sub-activity category, which is thought to be due to the large individual difference within a small sample, so that no meaningful change was detected. This needs to be reexamined in repeated studies using more samples in future. In a randomized controlled trial of a walking exercise program for Korean office workers in their 30s and 40s (mostly women), the level of physical activity, health-promoting behavior, and quality of life improved, constituting similar results to this study [16]. A systematic review of physical activity interventions for African American women found that a healthy diet, walking, and social support promoted physical activity [49], which also supports our findings. In the WEFHPP, online education and group interaction, which provided enough materials on a variety of lifestyle-related topics through MIM, seems to have been useful in facilitating a shift to healthy behaviors and lifestyles on the part of participants. The ongoing personal and social motivation strategies used in this study may also have contributed to adherence to health behaviors on the part of participants. Indeed, the acquisition of behavioral skills, use of mobile devices, and goal-setting and feedback are effective in increasing physical activity [50,51,52]. However, a randomized clinical trial of a lifestyle-focused physical activity program for middle-aged African American women found that group meetings greatly increased physical activity and prevented weight gain, but motivational telephone calls did not provide any additional benefit [52]. Further studies are needed to assess various interventional delivery strategies and to evaluate their effectiveness.

The WEFHPP reduced participants’ waist circumference (*p* < 0.001) but did not significantly change their BMI or blood pressure. In a meta-analysis of the effects of walking on cardiovascular risk factors in subjects of various sexes and ages (adults and older), walking improved body weight, BMI, waist circumference, and blood pressure [53]. Recent studies found that the effects of walking were greater under certain conditions. The blood pressure of participants with higher baseline systolic pressure decreased to a greater extent than that of the other participants after guided walking exercises had been performed for >6 months [54]. After completing a 24-week walking exercise program, the BMI, waist circumference, and systolic and diastolic blood pressure of obese middle-aged women decreased significantly [55]. Pedometer-based walking exercises for overweight adults involving >10,000 steps/day improved body weight, waist circumference, and blood pressure [56,57]. As stated above, our relatively short-term program did not improve the BMI or blood pressure of the healthy middle-aged women. However, physiological indicators may have been maintained within the normal range, thereby preventing weight gain and hypertension. Our program significantly reduced waist circumference, so it has the potential to improve body image or prevent abdominal obesity. Therefore, regular walking exercise in daily life can influence physical health when incorporated with other lifestyle factors such as overall physical activity, nutrition and stress management.

Finally, our program suggests the potential for improved mental health by reducing perceived stress (*p* < 0.001). Stults-Kolehmainen and Sinha (2014) confirmed a correlation between psychological stress and lack of physical activity in a meta-analysis of 168 papers [58]. Physical activity relieved stress, and stress was negatively associated with physical exertion. Additionally, an intervention combining stress management and exercise was proposed based on those findings [58]. Our WEFHPP relieved stress and increased physical activity in a synergistic manner. A short-term, randomized crossover trial combining walking and relaxation interventions for healthy adults reduced blood pressure and stress [59]. As such, focusing on walking exercise and stress management in primary care settings will be effective in achieving health promotion outcomes.

The main strength of our study is that it is based on a theoretical framework that can induce effective behavioral changes using a variety of strategies and behavioral skills that can be easily applied in daily life. This has the advantages of being easy to apply in practice, as well as for developing other interventions for different subjects and purposes. However, we enrolled a small number of participants, all from the same region; studies with larger and more diverse populations are needed. Our study also has limitations in that we did not sufficiently control for various exogenous variables (diet, smoking, antihypertensive drugs) that may have influenced the outcome variables. This should be considered in future studies using multidimensional outcome variables. New challenges for middle-aged women research were presented based on the Seattle Middle-aged Women’s Health Study, which analyzed longitudinal data spanning up to 23 years [60]. The aim was to go beyond the menopause-focused perspective to an in-depth exploration of life events and coping styles, as a way to find balance [60]. To understand the health and life of middle-aged women more fully, qualitative research on these topics is required in various cultures in future. Furthermore, because health-promoting behaviors should be sustained over the long term, to lead to health outcomes, a cohort or longitudinal study is needed to confirm the long-term effects of interventions.

## 6. Conclusions

We explored the effects of a walking exercise-focused health promotion program based on the IMB model for middle-aged women living in the community. The level of physical activity and health-promoting behavior increased, while waist circumference and perceived stress decreased. Our theory-based intervention has advantages in terms of high standardization potential, high availability, and improvement of health behaviors and health status. In future, this approach will be useful for devising interventions that meet the health needs of people concerned about their quality of life in the second half of life.

## Figures and Tables

**Table 1:** Contents of each session of the health promotion program.

Session	Health Information	Health Motivation		Behavioral Skills
1 ^†^	- Orientation: Program introduction, instructions on how to wear/use the pedometer and write an exercise log - Pretest: Filling out questionnaires, body measurement, blood pressure check			
	- Group health education (90 min): Proper walking and stretching, efficacy/calorie consumption of walking, health-promoting lifestyle, stress management	SGD	Continuous group support Pedometer monitoring Goal setting/ exercise log	Regular walking exercise (Correct walking for at least 30 min, 3 days a week)
2	- Health education using group MIM: Information on correct walking (frequency, posture, gaze, arm movements, stride length, gait sequence, and walking patterns, etc.)	IPC		
3	- Health education using group MIM: Exercise method and intensity tailored to the individual, considering maximum oxygen intake and maximum heart rate			
4	- Health education using group MIM: Stretching before/after exercise (video training)			
5	- Health education using group MIM: Nutrition: Proper eating tips, eating habits, etc.			
6^†^	- Group health education (40 min): Retraining on health-promoting lifestyle and stress management - Intermediate checking and resetting goals	SGD, IPC		
7	- Health education using group MIM: Stress management tips			
8	- Health education using group MIM: How to increase your daily activity			
9	- Health education using group MIM: Summary, sharing difficulties, Q&A	SGD		
10 ^†^	- Sharing experiences and rewarding for individual athletic performance - Post-test: Filling out questionnaires, body measurement, blood pressure check			

^†^ Off-line sessions in the program room. MIM = mobile instant messengers; SGD = small group discussion; IPC = individual phone counseling.

**Table 2:** Between-group comparisons of general characteristics and outcome variables at baseline (*N* = 44).

Variables	Categories	Exp. (*n* = 22)	Cont. (*n* = 22)	χ^2^, t, or Z	*p*
		*n* (%) or Mean ± SD			
Demographic characteristics					
Age (Range: 40–58)		48.05 ± 5.19	45.91 ± 5.20	8.200	0.916
Marital status	Married	18 (81.8)	21 (95.5)	2.031	0.172
	Unmarried	4 (18.2)	1 (4.5)		
Monthly income (KRW 10,000 = USD 7.86)	<300	12 (54.5)	12 (54.5)	0.000	0.619
	≥300	10 (45.5)	10 (45.5)		
Occupation	Office workers	4 (18.2)	2 (9.1)	2.696	0.610
	Service workers	10 (45.5)	7 (31.8)		
	Self-employed	2 (4.5)	2 (4.5)		
	Housewives	6 (27.3)	11 (50.0)		
Outcome variables					
Physical activity level (MET∙min)	Total score	887.24 ± 627.73	627.73 ± 588.34	−1.094	0.294
	Vigorous activity	160.00 ± 308.40	61.82 ± 212.28	−1.282	0.200
	Moderate activity	50.91 ± 86.57	130.91 ± 311.36	−0.660	0.509
	Walking activity	762.00 ± 1114.48	426.00 ± 483.36	−0.584	0.559
Health-promoting lifestyle	Total score	114.19 ± 16.59	116.41 ± 16.95	−0.441	0.662
	Health responsibility	17.46 ± 4.35	17.46 ± 3.71	0.000	1.000
	Physical activity	14.00 ± 4.45	13.09 ± 2.67	−0.567	0.571
	Nutrition	22.19 ± 3.20	21.86 ± 4.28	−0.498	0.619
	Spiritual growth	21.72 ± 4.20	22.54 ± 4.22	−0.645	0.523
	Interpersonal relations	21.14 ± 2.82	22.41 ± 4.03	−1.214	0.232
	Stress management	17.68 ± 3.67	19.05 ± 3.86	−1.201	0.237
Physiological indicators	Body mass index (kg/m^2^)	22.43 ± 2.32	21.57 ± 2.32	1.233	0.225
	Waist circumference (cm)	76.68 ± 6.83	74.91 ± 7.46	0.822	0.416
	Systolic blood pressure (mmHg)	117.18 ± 11.77	117.45 ± 10.57	−0.081	0.936
	Diastolic blood pressure (mmHg)	76.36 ± 8.18	76.55 ± 10.37	−0.343	0.732
Perceived stress		29.13 ± 4.06	27.59 ± 3.56	1.343	0.187

Exp = experimental group; Con = control group; SD = standard deviation.

**Table 3:** Between-group differences of outcome variables after intervention (*N* = 44).

Variables		Exp. (*n* = 22)	Cont. (*n* = 22)	t or Z	*p*
		Mean ± SD			
Physical activity level (MET∙min)					
Total score	Pretest	887.24 ± 725.80 ^†^	627.73 ± 588.34	−2.065	0.039
	Post-test	1606.27 ± 1124.14	853.16 ± 639.97		
	Difference	795.52 ± 1157.19	225.43 ± 585.96		
Vigorous activity	Pretest	160.00 ± 308.40	61.82 ± 212.28	−1.508	0.132
	Post-test	478.00 ± 642.16	160.00 ± 320.00		
	Difference	302.00 ± 533.58	98.18 ± 401.09		
Moderate activity	Pretest	50.91 ± 86.57	130.91 ± 311.36	−1.922	0.055
	Post-test	209.52 ± 239.97	150.91 ± 323.77		
	Difference	156.19 ± 217.22	20.00 ± 434.82		
Walking activity	Pretest	762.00 ± 1114.48	426.00 ± 483.36	−0.657	0.511
	Post-test	979.71 ± 776.82	537.71 ± 403.99		
	Difference	181.43 ± 826.73	111.71 ± 411.11		
Health-promoting lifestyle					
Total score	Pretest	114.19 ± 16.59	116.41 ± 16.95	3.344	0.002
	Post-test	134.91 ± 18.10	120.72 ± 17.69		
	Difference	20.73 ± 18.94	4.32 ± 13.08		
Health responsibility	Pretest	17.46 ± 4.35	17.46 ± 3.71	1.836	0.073
	Post-test	20.68 ± 4.41	18.18 ± 4.15		
	Difference	3.23 ± 5.06	0.73 ± 3.89		
Physical activity	Pretest	14.00 ± 4.45	13.09 ± 2.67	−3.601	<0.001
	Post-test	20.00 ± 4.09	13.77 ± 3.70		
	Difference	6.00 ± 4.67	0.68 ± 3.68		
Nutrition	Pretest	22.19 ± 3.20	21.86 ± 4.28	−2.758	0.006
	Post-test	26.00 ± 2.83	23.00 ± 4.81		
	Difference	3.82 ± 3.02	1.14 ± 2.83		
Spiritual growth	Pretest	21.72 ± 4.20	22.54 ± 4.22	1.493	0.143
	Post-test	23.95 ± 4.76	23.27 ± 3.58		
	Difference	2.23 ± 3.68	0.73 ± 2.95		
Interpersonal relations	Pretest	21.14 ± 2.82	22.41 ± 4.03	1.743	0.089
	Post-test	23.59 ± 3.10	23.14 ± 3.99		
	Difference	2.46 ± 3.31	0.73 ± 3.27		
Stress management	Pretest	17.68 ± 3.67	19.05 ± 3.86	2.623	0.012
	Post-test	20.68 ± 3.05	19.36 ± 3.71		
	Difference	3.00 ± 3.92	0.32 ± 2.77		
Physiological indicators					
Body mass index (kg/m^2^)	Pretest	22.43 ± 2.32	21.57 ± 2.32	−1.783	0.082
	Post-test	22.12 ± 2.27	21.55 ± 2.06		
	Difference	−0.32 ± 0.55	−0.02 ± 0.55		
Waist circumference (cm)	Pretest	76.68 ± 6.83	74.91 ± 7.46	−4.328	<0.001
	Post-test	74.59 ± 6.45	74.91 ± 7.32		
	Difference	−2.10 ± 1.77	0.00 ± 1.41		
Systolic blood pressure (mmHg)	Pretest	117.18 ± 11.77	117.45 ± 10.57	−0.757	0.453
	Post-test	113.45 ± 8.47	115.82 ± 11.94		
	Difference	−3.73 ± 9.75	−1.64 ± 8.54		
Diastolic blood pressure (mmHg)	Pretest	76.36 ± 8.18	76.55 ± 10.37	−0.875	0.382
	Post-test	76.86 ± 7.27	74.64 ± 9.29		
	Difference	0.50 ± 7.25	−1.91 ± 8.38		
Perceived stress					
	Pretest	29.13 ± 4.06	27.59 ± 3.56	−3.578	<0.001
	Post-test	23.09 ± 2.02	25.46 ± 3.25		
	Difference	−6.05 ± 4.56	−2.14 ± 2.34		

^†^ Off-line sessions in the program room. Exp = experimental group; Con = control group; SD = standard deviation.

## Data Availability

Please contact the corresponding author for data availability.
